# Efficacy of Stereotactic Body Radiotherapy in Patients With Hepatocellular Carcinoma Not Suitable for Transarterial Chemoembolization (HERACLES: HEpatocellular Carcinoma Stereotactic RAdiotherapy CLinical Efficacy Study)

**DOI:** 10.3389/fonc.2021.653141

**Published:** 2021-03-19

**Authors:** Thomas B. Brunner, Dominik Bettinger, Michael Schultheiss, Lars Maruschke, Lukas Sturm, Nico Bartl, Ivana Koundurdjieva, Simon Kirste, Hannes P. Neeff, Christian Goetz, Nils Henrik Nicolay, Gabriele Ihorst, Fabian Bamberg, Robert Thimme, Anca-Ligia Grosu, Eleni Gkika

**Affiliations:** ^1^Department of Radiation Oncology, University Medical Center Magdeburg, Magdeburg, Germany; ^2^Berta-Ottenstein-Programme, University of Freiburg, Freiburg, Germany; ^3^Department of Medicine II, Medical Center-University of Freiburg, Freiburg, Germany; ^4^Department of Radiology, University Medical Center Freiburg, Freiburg, Germany; ^5^Department of Radiation Oncology, Medical Center - University of Freiburg, Freiburg, Germany; ^6^Department of General and Visceral Surgery, University Medical Center Freiburg, Freiburg, Germany; ^7^Department of Nuclear Medicine, University Medical Center Freiburg, Freiburg, Germany; ^8^German Cancer Consortium (DKTK) Partner Site Freiburg, German Cancer Research Center (DKFZ), Heidelberg, Germany; ^9^Faculty of Medicine, University of Freiburg, Freiburg, Germany; ^10^Clinical Trials Unit Freiburg, Faculty of Medicine, University of Freiburg, Freiburg, Germany

**Keywords:** hepatocellular carcinoma, radiotherapy, stereotactic body radiation therapy, Transarterial Chemoembolization, hepatocellar carcinoma

## Abstract

The aim of this prospective observational trial was to evaluate the efficacy, toxicity and quality of life after stereotactic body radiation therapy (SBRT) in patients with hepatocellular carcinoma (HCC) and to assess the results of this treatment in comparison to trans-arterial chemoembolization (TACE). Patients with HCC, treated with TACE or SBRT, over a period of 12 months, enrolled in the study. The primary endpoint was feasibility; secondary endpoints were toxicity, quality of life (QOL), local progression (LP) and overall survival (OS). Between 06/2016 and 06/2017, 19 patients received TACE and 20 SBRT, 2 of whom were excluded due to progression. The median follow-up was 31 months. The QOL remained stable before and after treatment and was comparable in both treatment groups. Five patients developed grade ≥ 3 toxicities in the TACE group and 3 in the SBRT group. The cumulative incidence of LP after 1-, 2- and 3-years was 6, 6, 6% in the SBRT group and 28, 39, and 65% in the TACE group (*p* = 0.02). The 1- and 2- years OS rates were 84% and 47% in the TACE group and 44% and 39% in the SBRT group (*p* = 0.20). In conclusion, SBRT is a well-tolerated local treatment with a high local control rates and can be safely delivered, while preserving the QOL of HCC patients.

## Introduction

The incidence and mortality of hepatocellular carcinoma (HCC) is rising worldwide due to the rise of viral hepatitis and non-alcoholic steatohepatitis (NASH) (1). The overall 5-year survival rate is 5% with more than 70% of the patients presenting with advanced disease precluding curative treatment such as liver transplantation, resection, or local ablative treatments such as radiofrequency ablation (RFA) or microwave ablation (2). According to the current Barcelona Clinic Liver Cancer (BCLC) classification for patients who are not eligible for resection or liver transplantation, treatment options include local non-surgical methods such as RFA, trans-arterial chemoembolization (TACE), and systemic therapy (3). Unfortunately, in about 15–20% of the patients who would benefit from local therapy, none of those treatments can be offered, due to the respective limitations and contraindications, such as decompensated cirrhosis, tumor extension, severely reduced portal flow, renal insufficiency (3). For these patients, SBRT could be offered as an alternative local ablative therapy with high rates of local control (4), while maintaining a good quality of life (5, 6). To date, there are no published prospective randomized trials comparing SBRT with TACE in locally advanced HCC, as these trials are ongoing (NCT02470533, NCT03326375 NCT03338647 NCT03338647). The aim of this study was to assess the feasibility of SBRT in everyday clinical practice, in patients with HCC, prior to a randomized trial.

## Methods and Materials

This is a prospective, two-arm, non-randomized, study analyzing the role of SBRT and TACE in patients with HCC. The primary objective of this trial was to investigate the feasibility of SBRT in everyday clinical practice, prior to a randomized trial. Secondary endpoints were: toxicity according to the NCI-CTCAE v4.0 for adverse events, health related quality of life (QOL), incidence of local progression (LP) (according to mRECIST), overall survival (OS) and progression free survival (PFS). The active recruitment time was 12 months. This study was registered at the German Clinical Trials Register (DRKS 00008566) and was approved by the local ethics committee (374/15).

### Treatment

Patients received either TACE or SBRT according to the decision of the institutional HCC tumor board, taking into account the standard treatment algorithm (3). TACE was offered in patients with localized disease and/or with contraindications for resection, transplantation or RFA. For patients where TACE or systemic treatment were not deemed suitable either due to exclusion criteria (Supplementary Table 1) or for any other reason such as patient preference, SBRT was offered as an alternative.

### TACE

The procedure involved gaining percutaneous trans-arterial access by the Seldinger technique to the hepatic artery with an arterial sheath, usually by puncturing the common femoral artery in the right groin and passing a catheter guided by a wire through the abdominal aorta, through the celiac trunk and common hepatic artery, and finally into the branch of the proper hepatic artery supplying the tumor. Afterwards a selective angiogram of the celiac trunk and in specific situations additionally of the superior mesenteric artery was performed in order to identify the branches of the hepatic artery supplying the tumor(s). This was done to maximize the amount of the chemotherapeutic dose that is directed to the tumor and minimize the amount of the chemotherapeutic agent that could damage the normal liver tissue. When a tumor supplying blood vessel was selected, alternating aliquots of the chemotherapy (epirubicin or mitomycin (doses of max. 100 mg) and of embolic particles, or particles containing the chemotherapy agent, were injected through the catheter. The total chemotherapy dose was given into a single vessel, or divided among several vessels supplying the tumor/s. Patients were discharged from hospital several hours after the end of the procedure or on the following day. Re-staging CT scans were performed in accordance to clinical practice 12 weeks after TACE if complete embolization was achieved. In case of incomplete treatment and therefore tumor persistence TACE was repeated in a 4 week interval.

### SBRT

Patients underwent a 4D and multiphase CT (arterial phase and/or delayed phase and venous phase), using a custom immobilization (e.g., vacuum cushion immobilization, patient positioning boards, knee cushions, and abdominal compression).

The primary tumor(s) and any tumor vascular thrombus, if present, were included into the gross tumor volume (GTV). For all patients, image guided radiotherapy (IGRT) using cone beam CT (CBCT) for every fraction were mandatory and if necessary for IGRT, fiducial marker implantation was performed prior to planning CT.

Treatment was delivered in 3, 5, 8, or 12 fractions. A total dose of 45 Gy in 3 fractions, 50 Gy in 5 fractions, 60 Gy in 8 fractions or of 66 Gy in 12 fractions, aiming to achieve a biological effective dose (BED) of close to 100 Gy (α/β = 10 Gy). The number of fractions was chosen based on the volume of normal tissues irradiated, considering the dose constraints for organs at risk such as stomach, duodenum, small and large bowel and liver. Dose prescription was chosen so that 95% of the PTV received at least the nominal fraction dose, and 99% of the PTV received a minimum of 90% of the nominal dose (according to ICRU 83). The dose maximum within the PTV was 110–120% of the prescribed dose. Sub-volumes close to critical OARs were allowed to receive a lower dose to avoid toxicities, using a simultaneous integrated protection (SIP) (7).

### Response Evaluation

Treatment response was evaluated using the international criteria proposed in the Reviewed Response Evaluation Criteria in Solid Tumors (mRECIST) Guideline version 1.1 (8) from a panel of an experienced radiologist and radiation oncologist. For patients treated with TACE, tumors requiring multiple embolization procedures because of residual disease were not counted as failures. Response assessments including response of tumor thrombosis, physical examination and blood tests (such as complete blood counts and biochemical analysis including liver function) were repeated every 3 months.

### Quality of Life Assessment

Patients treated with SBRT filled in the EORTC QLQ-C30 and QLQ C29 at the first treatment, 4 weeks after the last treatment and at the second follow up (3 months later). For the patients treated with TACE the QLQ assessment was before and after the treatment.

### Statistical Methods

All analyses were based on the assigned treatment arm for all eligible patients for whom treatment was started. Continuous variables are reported as median with the corresponding range (minimum and maximum), and categorical variables are presented as absolute and relative frequencies unless stated otherwise. Baseline group comparisons were conducted with Fisher's exact test or the Chi-square test (binary variables) or Wilcoxon's two-sample test (continuous variables) as a normal distribution was questionable for the respective variables.

OS was calculated as time from start of treatment until death from any cause, with censoring at the date last seen alive. PFS was calculated as time from start of treatment until death or documentation of progression. PFS times were censored at the date patients were last seen alive without documentation of disease progression. PFS times were censored in case that observation of death was more than 3 months after the last documented response assessment, in line with FDA recommendations (FDA Guidance for Industry, Clinical Trial Endpoints for the Approval of Cancer Drugs and Biologics, May 2007). OS and PFS were estimated by the Kaplan-Meier method. The comparison of the two arms using log-rank tests was regarded as descriptive information. The Cox proportional hazards regression model was used for further analyses of possible prognostic factors for OS and PFS. The small number of patients did not allow complex multivariate modeling with variable selection using forward selection. Therefore, variables considered in a multivariate model were selected according to (i) large baseline differences between SBRT and TACE patients, (ii) relevant univariate impact on OS or PFS. A forward variable selection approach was then applied. Locally controlled survival (LCS) was defined as time to local progression or death, with censoring in the same manner as described for PFS. Analyses were performed with the Kaplan-Meier method and the log-rank test. The components of this combined endpoint LCS were analyzed separately under consideration of competing events. Thus, local progression (LP) was estimated as cumulative incidence rates taking into account that death without prior documentation of local disease progression is a competing event that prevents the observation of local progression.

Estimation of the effects of possible prognostic factors for LP was done with the Fine and Gray regression model. Results are presented as sub-distribution hazard ratios with 95% confidence intervals. Analyses were performed with SAS V9.4 (SAS Institute Inc., Cary, NC, USA).

## Results

### Patient Characteristics

**Figure 1:**
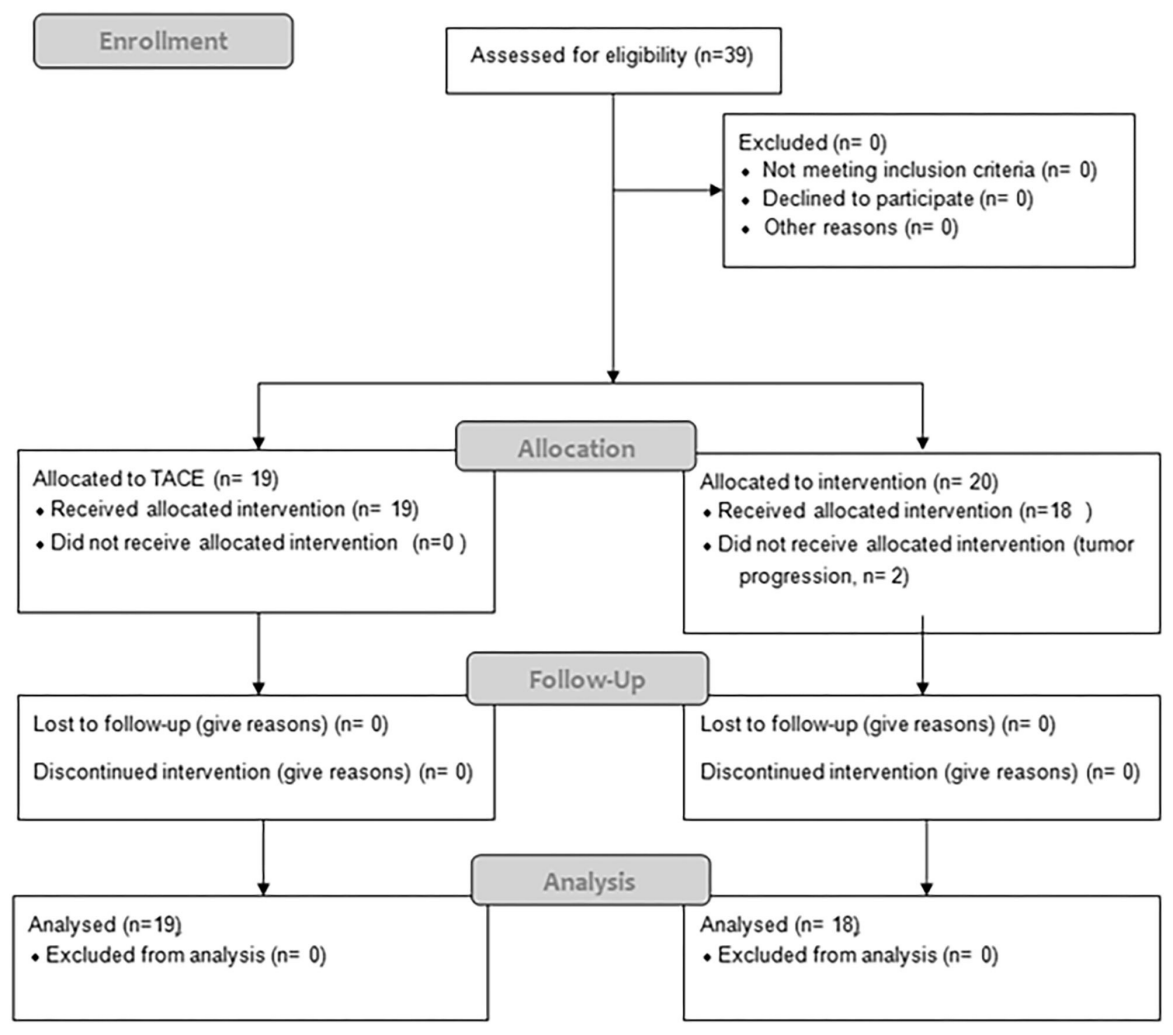
Consort diagram.

Between 06/2016 and 06/2017, 19 patients received TACE and 20 patients were planned for SBRT; however SBRT was halted in two patients due to progression (Figure 1), resulting in 18 evaluable patients. In general patients treated with SBRT were older (76 vs. 69, *p* = 0.36), had larger tumors (median 42 vs. 32 cm, *p* = 0.08) and higher BCLC stages (*p* = 0.0013). Additionally 3 patients (17%) had a metastatic disease (lung *n* = 2, adrenal *n* = 1), and a 6 patients (37%) a portal vein thrombosis (PVT), all in the SBRT arm. Seven patients (37%) in the TACE arm had HCC-directed therapy prior to enrolment and 11 (61%) in the SBRT arm (most of them had > 1 treatment modalities). The median time between diagnosis and treatment was 1 month (range: 0–28) in the TACE group and 3.5 months (range: 1–98) in the SBRT group. Ten patients (53%) in the TACE group received further treatments and 8 patients (44%) in the SBRT group. Patient and treatment characteristics are shown in Table 1.

**Table 1:** Patient and treatment characteristics.

	**TACE**	**SBRT**	*p*-value^*^
**A. PATIENT CHARACTERISTICS**			
Age			0.36
median (range)	69 (45–92)	76 (58–85)	
**Gender**			
Male	18 (95%)	13 (72%)	0.09
Female	1 (5.3%)	5 (28%)	
Etiology of liver disease			0.74
HBV	2 (11%)	4 (22%)	
HCV	5 (23%)	5 (28%)	
Alcohol induced	2 (11%)	6 (33%)	
NASH	2 (11%)	1 (6%)	
n.a.	8 (42%)	2 (11%)	
Treatments before study inclusion^†^			0.19^**^
Resection	0 (0%)	4 (22%)	
RFA	2 (11%)	0 (0%)	
TACE	7 (26%)	11 (61%)	
SBRT	0 (0%)	1 (6%)	
Sorafenib, regorafenib	0 (0%)	0 (0%)	
SIRT	0 (0%)	2 (11%)	
No prior treatments	12 (63%)	7 (39%)	
Further treatments after study inclusion^†^			0.6
Resection	1 (5%)	0 (0%)	
Transplantation	1 (5%)	0 (0%)	
TACE	6 (32%)	2 (11%)	
SBRT	2 (11%)	3 (17%)	
Sorafenib, regorafenib	2 (11%)	5 (28%)	
SIRT	0 (0%)	1 (6%)	
No further treatments	9 (47%)	10(56%)	
BCLC			0.0013
A	7 (37%)	2 (11%)	
B	12 (63%)	7 (40%)	
C	0 (0%)	9 (50%)	
D	0 (0%)	0 (0%)	
Metastatic disease	0 (0%)	3 (17%)	0.10
Child pugh score baseline			0.40^***^
A	17 (90%)	14 (78%)	
5	15	8	
6	2	6	
B	2 (10%)	4 (22%)	
7	1	2	
8	1	2	
ALBI grade			0.51
1	9 (47%)	11(61%)	
2	10 (53%)	7 (40%)	
3	0 (0%)	0 (0%)	
Maximal tumor diameter (median, range), mm	32 (10–78)	42 (21–210)	0.08
Portal vein thrombosis (PVT)	0 (0%)	6 (37%)	0.01
**B. TREATMENT CHARACTERISTICS**			
cTACE			8 (42%)
Drug-eluting beads TACE			11 (58%)
**Total number of TACE sessions**			
One TACE			6 (32%)
Two TACE			7 (37%)
Three TACE			2 (11%)
Five TACE			1 (5%)
Six TACE			1 (5%)
**SBRT**			
Total prescribed dose, median (IQR)			55 (49–60) Gy
BED_10_, median (IQR)			100 (75–139) Gy
Dose per fraction, median (IQR)			7.2 (5–15.9) Gy
Nr of fractions, median (IQR)			5 (3–12)

cTACE, conventional transarterial chemoembolization; BED, biological effective dose; NASH, non -alcoholic steatohepatitis; BCLC, Barcelona Clinic Liver Cancer; TACE, trans-arterial chemoembolization; PVT, portal vein thrombosis; SBRT, stereotactic body radiation therapy; cTACE, conventional transarterial chemoembolization;

* Fisher's exact test (binary variables) or Wilcoxon's two-sample test (continuous variables);

** yes vs. no prior treatment;

*** *A vs. B; n.a, not available*.

† *some patients received multiple treatments*.

### Quality of Life

Patients in the SBRT group had a worse, but not statistically significant, QOL at baseline compared to the TACE group (Supplementary Figure 1, Supplementary Tables 2A–C). After treatment there was a slight, but not statistically significant, improvement in the QOL between baseline and 1st follow up in the SBRT group (Supplementary Table 2) and there was no difference between the pre- and post-TACE quality QOL (Supplementary Table 2).

### Toxicity

Toxicities were moderate in both groups. Three (17%) patients developed grade ≥ 3 toxicities in the SBRT group, two patients developed grade 3 hepatic failure with grade 3 bilirubin increase in the SBRT group and one patient developed a grade 5 fistula. In the TACE group, 5 (26%) patients developed grade ≥ 3 toxicities. One patient developed a grade 3 bilirubin increase, grade 4 aspartat aminotransferase (AST) and alanine aminotransferase (ALT) increase grade 3 pain and grade 5 hepatic failure. One patient developed grade 4 bilirubin increase, grade 3 AST increase, grade 3 ascites and grade 3 hepatic failure. One patient developed a grade 4 ALT increase, a grade 3 AST increase, a grade 3 pancreatitis and a grade 5 liver abscess. Another patient developed a grade 4 bilirubin increase, grade 3 cholangitis and grade 3 hepatic failure and one patient developed a grade 4 GPT and GOT increase. There was no statistical significant difference (*p* = 0.69, fisher's exact test) in the incidence of toxicities between the two groups. Albumin-bilirubin (ALBI) grade, Child Pugh (CP) score and blood test changes over time are shown in Supplementary Tables 3, 4.

### Local Progression, Progression Free Survival, and Overall Survival

**Figure 2:**
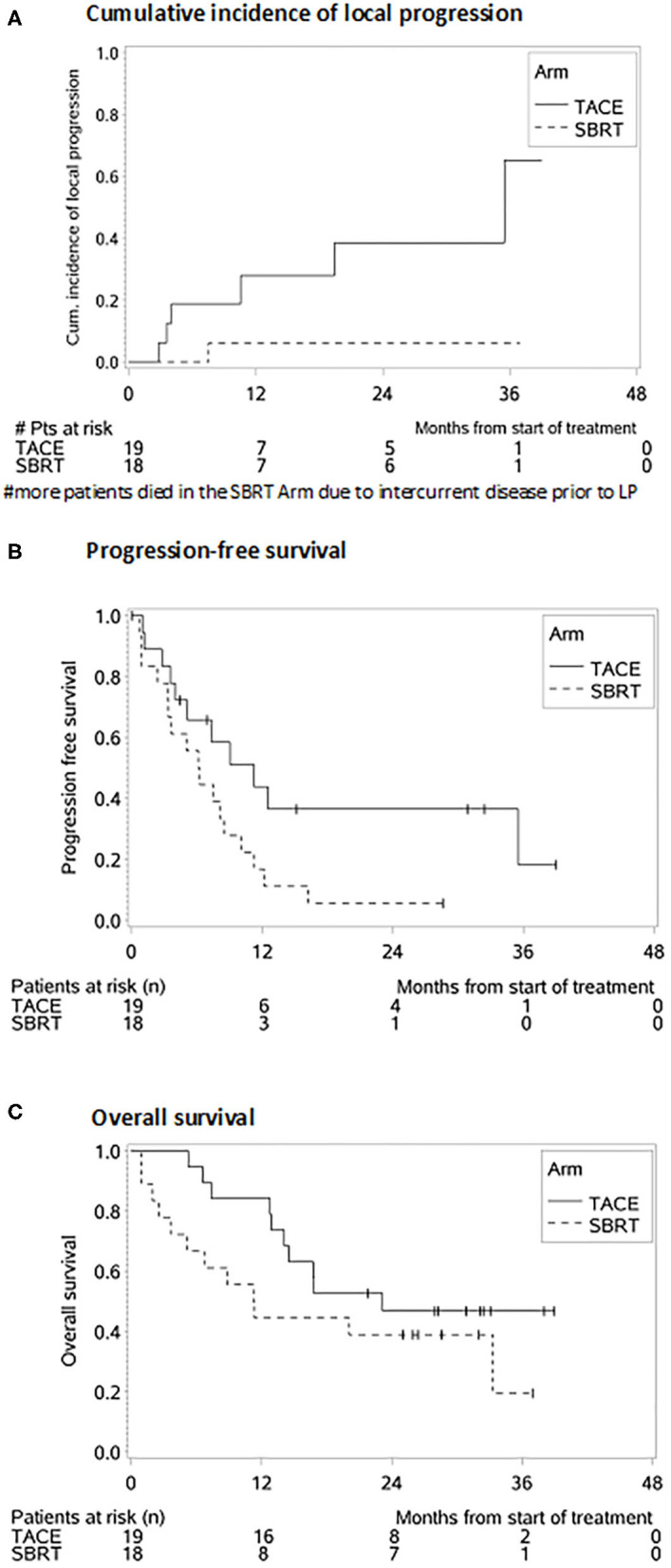
Survival probabilities after SBRT and TACE. **(A)** Cumulative incidence of local progression. **(B)** Progression-free survival. **(C)** Overall survival.

The cumulative incidence of LP after 1-, 2-, and 3-years (where death without prior LP was defined as a competing event) was 6, 6, and 6% in the SBRT arm and 28, 39, and 65% in the TACE arm (*p* = 0.02 Gray test) (Figure 2A). The observation of LP might have been prevented in the SBRT arm due to a higher incidence of intercurrent deaths within the first 3 months. No other factors except for the type of the treatment (HR 0.119, 0.015–0.993, *p* = 0.04 Fine and Gray regression model) were statistically significant concerning LP in univariate analysis (Table 2). The cumulative incidence rate of death (competing event) without prior documentation of LP after 1-, 2-, and 3 years were 52% in the SBRT group and 8% in the TACE group. The LCS (i.e., the time to LP or death) after 1-, 2-, and 3 years was 42.2, 42.2, and 42.2% in the SBRT group and 64, 53.3, and 26.7% in the TACE group (*p* = 0.42).

**Table 2:** Univariate fine and gray regression model for local progression.

**Parameter**	**HR**	**95% CI**	***p***
SBRT vs. TACE	0.119	0.015–0.933	0.04
ALBI grade (2–3 vs. 1)	0.222	0.029–1.702	0.15
Nodule (multiple vs. solitary)	2.602	0.557–12.154	0.22
Prior treatments (yes vs. no)	2.028	0.496–8.288	0.32
Tumor diameter ≥ 50 mm (yes vs. no)	0.581	0.129–2.614	0.48
BCLC (A vs. C)	1.438	0.094–22.086	0.79
BCLC (B vs. C)	3.040	0.385–24.022	0.29

*CI, confidence interval; HR, Hazard ratio; TACE, transarterial chemoembolization; SBRT, stereotactic body radiation therapy; ALBI, albumin bilirubin grade; HCC, hepatocellular carcinoma, BCLC, Barcelona liver clinic classification*.

The median PFS was 4 months in the SBRT group and 11 months in the TACE group (HR: 2.172, 95% CI 0.988–4.775, *p* = 0.05, Figure 2B) which remained also significant on multivariate analysis (HR: 2.855, 95% CI: 1.227–6.644, *p* = 0.02). Patients with a BCLC stage A (HR: 0.208, 95% CI: 0.055–0.787, *p* = 0.02), with multiple HCC (HR: 2.759, 95% CI: 1.207–6.3006, *p* = 0.02) as well as patients with prior treatments (HR: 2.693, 95% CI: 1.199–6.046, *p* = 0.02) had a better PFS (Table 3).

**Table 3:** Univariate and multivariate Cox-regression analysis of overall survival.

	**Overall survival**			**Progression free survival**		
**Parameter**	**HR**	**95% CI**	***p***	**HR**	**95% CI**	***p***
**UNIVARIATE ANALYSIS**						
SBRT vs. TACE	1.716	0.739–3.985	0.21	2.172	0.988–4.775	0.05
Child pugh score (7–9 vs. 5–6)	3.968	1.419–11.096	0.01	1.831	0.689–4.864	0.23
ALBI grade (2–3 vs. 1)	1.677	0.724–3.587	0.23	1.181	0.551–2.531	0.67
nodules (multiple vs. solitary)	1.530	0.653–3.587	0.33	2.759	1.207–6.306	0.02
Prior treatments (yes vs. no)	1.286	0.554–2.982	0.56	2.693	1.199–6.046	0.02
Metastases (yes vs. no)	1.728	0.499–5.980	0.39	2.260	0.634–8.052	0.21
Diameter ≥ 50 mm (yes vs. no)	3.214	1.355–7.624	0.01	1.740	0.782–3.873	0.18
BCLC (A vs. C)	0.514	0.161–1.638	0.26	0.208	0.055–0.787	0.02
BCLC (B vs. C)	0.478	0.177–1.289	0.15	0.471	0.197–1.126	0.09
Portal vein thrombosis (yes vs. no)	1.454	0.626–3.374	0.03	2.341	0.919–5.965	0.07
**MULTIVARIATE ANALYSIS**						
SBRT vs. TACE	1.948	0.778–4.879	0.15	2.855	1.227–6.644	0.02
Child pugh score (7–9 vs. 5–6)	8.866	2.355–33.376	<0.01	5.637	1.661–19.123	<0.01
Diameter ≥ 50 mm (yes vs. no)	4.695	1.810–12.177	<0.01			
HCC (multiple vs. solitary)	3.344	1.171–9.547	0.02	5.021	1.840–13.699	<0.01

*CI, confidence interval; HR, Hazard ratio; TACE, transarterial chemoembolization; SBRT, stereotactic body radiation therapy; ALBI, albumin bilirubin grade; HCC, hepatocellular carcinoma, BCLC, Barcelona liver clinic classification*.

The median OS, in the TACE group was 23 vs. 11 months in the SBRT group and the 1 and 2 years OS rates 84% and 47% in the TACE arm and 44% and 39% in the SBRT arm, respectively (*p* = 0.20, Figure 2C) Three patients in the SBRT arm died within 1 month after completion of therapy due to pneumonia, urosepsis and sepsis due to necrotizing fasciitis after hip-endoprosthesis. Patients with a higher CP score (HR 3.968, 95% CI: 1.419–11.096, *p* = 0.01) larger tumors (HR: 3.214, 95% CI: 1.355–7.624, *p* = 0.01) and PVT (HR: 3.107, 95% CI: 1.116–8.648 *p* = 0.03) had a worse OS, which remained significant on multivariate analysis (Table 2).

## Discussion

Over the past 10 years there have been significant advances in the treatment of HCC. Although in patients with BCLC stage B TACE appears to be the treatment with the best quality of evidence leading to an improvement of the OS, in advanced HCC, which poses a more heterogeneous group, the selection of treatment type depends on many factors such as the performance status of the patient, the underlying cirrhosis, the presence of metastases or the extent of macrovascular extension (9). To date there are no published results on randomized trials comparing SBRT with TACE in HCC, as randomized studies are still ongoing. This is the first prospective trial, including both treatment options, TACE and SBRT, avoiding randomization on the purpose of reflecting clinical needs prior to a randomized trial.

**Figure 3:**
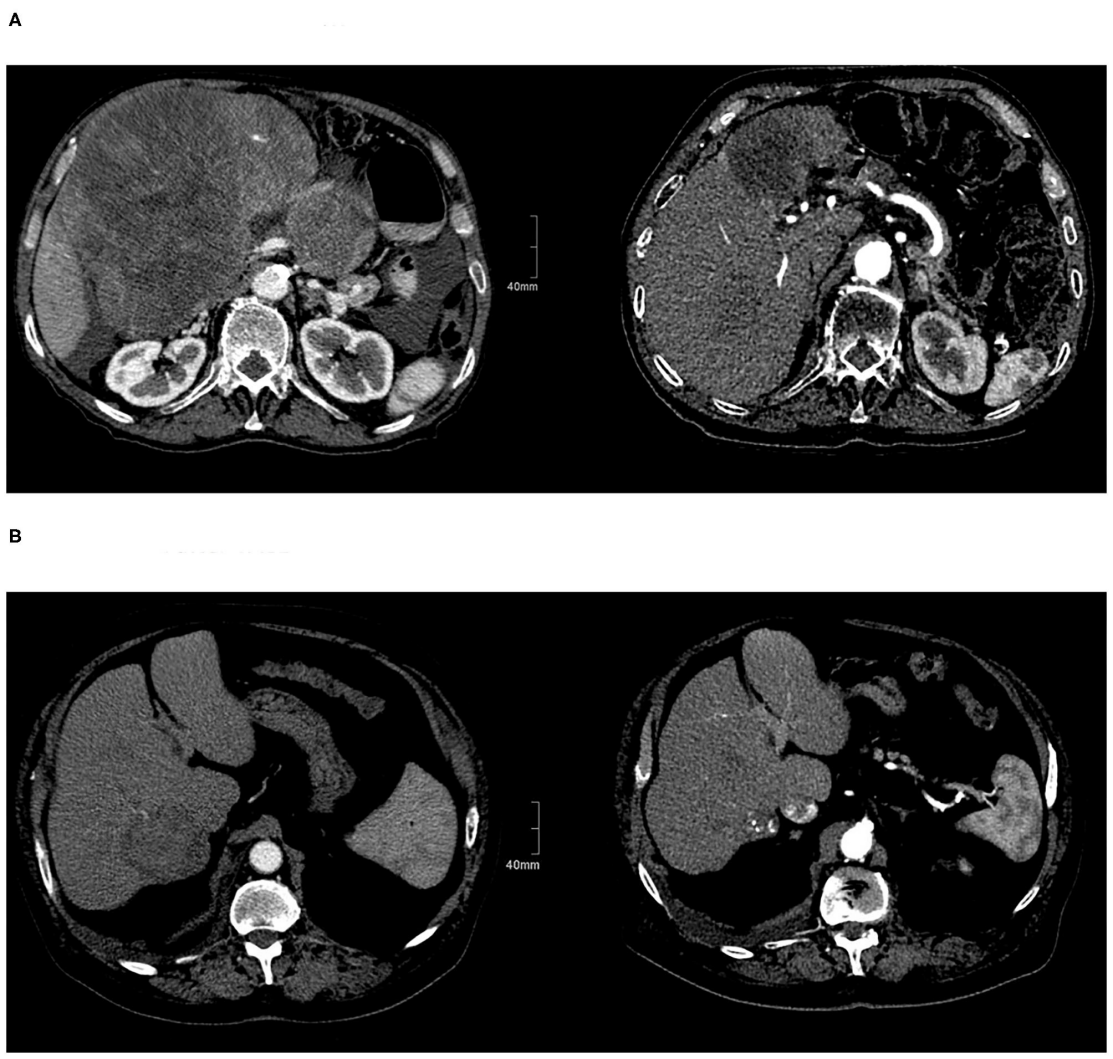
Patients with HCC before and after treatment. **(A)** Before SBRT and after SBRT. **(B)** Before and after TACE.

In the current prospective trial, patients in the SBRT arm were multi-morbid with advanced tumors and worse quality of life at baseline, not eligible for other treatments (Figure 3). Nevertheless, treatment was well-tolerated, while maintaining at least a stable QOL in the longitudinal assessment, independent of the comorbidities, and was similar for both SBRT and TACE. Similar results concerning the QOL after liver SBRT were also reported by Mendez Romero et al. (6) and Klein et al. (10) who observed a temporary worsening of appetite and fatigue, which was quickly resolved, resulting to an overall stable QOL, but there are no data comparing both treatments. Thus, patients ineligible for other local or system treatments tolerate the SBRT without impairing the QOL similar to patients with less advanced disease treated with TACE.

Additionally, the cumulative incidence of LP after 3-years of 6% in the SBRT group was high, similar to published literature, ranging between 64 and 96%, and 2-year OS rates ranging between 40 and 81% (11–20), corroborating the ablative potential of SBRT. Nevertheless, our results should be interpreted with caution under the consideration that in the 1st year, more deaths occurred in the SBRT group (as patients included in the SBRT arm were treatment-refractory and/or ineligible for other treatments) preventing the observation of LP. This explains also the difference in the OS and PFS between the both groups and the modest OS and PFS in the SBRT arm. Although there was no statistically significant difference in the OS the PFS was statistically significant better in the TACE group probably due to patient selection, as patients in the SBRT had more advanced tumors. In our study TACE was also well-tolerated in terms of QOL and toxicity leading to a median OS of 26 months similar to published literature (21, 22) with a low incidence of LP. Due to the lack of randomization in our study, a direct comparison between SBRT and TACE is not possible, but both treatments show high efficacy.

Several, retrospective, studies that used propensity score matching in order to compare both treatments, indicate that SBRT could be an alternative to TACE in terms of local control. Bettinger et al. (23) showed similar local control rates after 1 year (TACE: 82%, SBRT 84.8%, *p* = 0.8) and OS (TACE: 11 months, SBRT 9 months, *p* = 0.49) for both treatments, with moderate toxicity, whereas Sapir et al. (24) showed that both LC at 1 year (SBRT 91%, TACE 47%, *p* < 0.001) and toxicity (TACE: 13%, SBRT: 8%, *p* = 0.05) favored SBRT, without any difference in the OS. Similarly, in a study by Shen et al. (25) SBRT showed a better in-field control after 3 years (77.5 vs. 55.6%) and OS rates (3-year OS of 55 vs. 13%) than TACE in patients with medium-sized HCC, particularly for recurrent cases. But also in comparison to RFA, Wahl et al. (26), using propensity score analysis, observed a similar freedom from LP (FFLP) 83.8 vs. 80.2% at 2 years, while for tumors >2 cm, there was a decreased FFLP for RFA compared with SBRT (HR, 3.35; *P* = 0.025). In a another study, using propensity score matching in a cohort of 2,064 patients, after adjusting for clinical factors, SBRT was related to a significantly lower risk of local recurrence compared to RFA in both the entire (HR: 0.45, *p* < 0.001) and matched (HR 0.36, *p* < 0.001) cohorts (27). Similar results were also reported in a meta-analysis from Lee et al. (28) (SBRT vs. RFA: 84.5 vs. 79.5% *p* = 0.431). Yet, pooled analysis of OS in HCC studies showed an odds ratio of 1.43 (95% CI: 1.05–1.95, *p* = 0.023), favoring RFA. Additionally, radiotherapy shows similar results for TACE and RFA as bridging therapy (29). Of course, in the absence of prospective trials these results should be interpreted with caution, as some confounding cannot be ruled out, which could result in subtle biases.

Thus, SBRT is according to the NCCN guidelines (v5. 2020) reported not only as an alternative for patients ineligible for other local treatments, but as a treatment option a priori equal to other local treatments, but due to the lack of randomized trials, SBRT is not yet included in the current Barcelona Clinic Liver Cancer (BCLC) classification (30).

But also, in more advanced stages, SBRT showed an OS benefit as compared to sorafenib in highly selected patients. Using two international cohorts of patients (*n* = 1,023) treated with sorafenib, Bettinger et al. (31), found in a propensity score analysis that patients treated with SBRT had an improved OS compared to sorafenib (17 vs. 10 months, *p* = 0.012). The rationale for taking an aggressive approach to treating large liver tumors is that patients often die from liver failure related to disease progression regardless of the presence of extrahepatic disease (32). The clinical benefit of any local treatment option depends on the effectiveness of the modality and the a priori probability that LP will lead to mortality (32).

Currently checkpoint inhibitors play an increasingly important role in the treatment of several metastatic solid tumors as well as for primary liver tumors (33, 34). Ionizing radiation, apart from cytotoxicity, has been shown to additionally induce immune-modulatory effects, which trigger anti-tumor immune responses (35–40). SBRT, by applying a high single dose with a few but more than one fractions, seems to have the potential to lead to an activation of specific T-cell response in the tumor (41, 42). In pre-clinical models the most potent abscopal effects have been observed when CTLA4-anatagonist treatment was applied during RT with 3X8Gy (vs. 5X6 Gy) in breast and colon cancer-bearing mice and not with a single dose of 20 or 30 Gy (42, 43). Grassberger et al. (44), reported on circulating immune cell populations in response to stereotactic body radiation therapy in patients with liver cancer showing that the fraction of activated mid-treatment CTLs was significantly associated with OS. Thus, there is a rationale for combining immunotherapies (IT) with RT as the radiation induced immune activation of CTLs can be boosted by checkpoint inhibitors.

Our study has several limitations such as the small sample size, the fact that some patients in the SBRT arm died shortly after treatment and the lack of a direct randomization between both arms so that a direct comparison is not possible. Patients in both arms received a number of subsequent treatments, ranging from transplantation and resection to systemic treatment and best supportive care which interfered with the outcome in both arms, especially in the SBRT arm were patients received more treatments. Additionally a few patients had metastatic disease in the SBRT arm which is a negative bias.

Moreover, statistical differences were revealed between the two groups, in terms of BCLC stage and portal vein thrombosis. However, due the limited sample, it is likely that other types of variability exists, for example the presence of metastatic disease in SBRT group but not in TACE group, or range of tumor diameter. Furthermore, 42 % of the patients in the TACE arm were treated with conventional TACE, which might lead to a poorer survival, although DEB-TACE has not been shown to improve OS compared to conventional TACE in randomized trials or meta-analysis (9, 45, 46).

This is the first published trial evaluating TACE and SBRT in a prospective manner, showing that SBRT is a well-tolerated locally effective treatment that does not impair the quality of life of multi-morbid patients, and could be considered as an alternative in carefully selected patients with contraindications for TACE.

## Data Availability Statement

The original contributions presented in the study are included in the article/Supplementary Material, further inquiries can be directed to the corresponding author/s.

## Ethics Statement

The studies involving human participants were reviewed and approved by Ethics Committee University of Freiburg. The patients/participants provided their written informed consent to participate in this study.

## Author Contributions

TB, DB, MS, LM, A-LG, and EG: conceptualization. DB, MS, NB, IK, and EG: data curation. LM, GI, and EG: formal analysis. A-LG: funding acquisition. TB, DB, MS, LM, LS, NB, SK, HN, CG, FB, RT, A-LG, and EG: investigation. TB, DB, MS, LM, A-LG, and EG: methodology. TB, DB, MS, LM, and EG: project administration. TB, DB, LM, LS, NB, SK, HN, CG, FB, RT, and EG: resources. TB, MS, NN, and GI: software. TB, A-LG, and EG: supervision. GI: validation. GI and EG: visualization. TB, DB, MS, FB, NN, GI, A-LG, and EG: writing – original draft. TB, DB, MS, LM, LS, NB, IK, SK, HN, CG, NN, GI, FB, RT, A-LG, and EG: writing – review & editing. All authors contributed to the article and approved the submitted version.

## Conflict of Interest

DB: consulting and advisory: Bayer Healthcare, Boston Scientific; teaching and speaking fees: Falk Foundation. MS: consulting and advisory: Bayer Healthcare; teaching and speaking fees: L.W. Gore, Falk Foundation. The remaining authors declare that the research was conducted in the absence of any commercial or financial relationships that could be construed as a potential conflict of interest.
