# Supplementary material for: Efficacy of Stereotactic Body Radiotherapy in Patients With Hepatocellular Carcinoma Not Suitable for Transarterial Chemoembolization (HERACLES: HEpatocellular Carcinoma Stereotactic RAdiotherapy CLinical Efficacy Study)
Source: Front Oncol. 2021 Mar 19;11:653141. doi: 10.3389/fonc.2021.653141 (PMC8017336; doi:10.3389/fonc.2021.653141)
Supplement: Supplementary file 1 [file Data_Sheet_1.docx]

Supp. Table 1- Inclusion/Exclusion Criteria

| Inclusion Criteria |
| --- |
| A: SBRT Group |
| Age ≥ 18 years, male and female  Histologically or cytologically confirmed hepatocellular carcinoma OR diagnosis made with characteristic enhancement in 4-Phase CT or MRI corresponding to AASLD- / EASL guidelines in cirrhotic patients.  Discussion in a routine multidisciplinary tumour board  Patients unsuitable for surgery, TACE*, RFA, or alcohol ablation  Understanding of procedure, significance and consequences of the study  signed informed consent  bilirubin has to be < 4 x the upper limit of normal, AST or ALT < 6 x the upper limit of normal, international normalized ratio < 1.5 except if patients are on oral anticoagulation, haemoglobin≥ 90 g/L, platelets ≥ 50 x 10^9^/L, and neutrophils ≥ 1.0 x 10^9^/L |
| B: TACE Group |
| Age ≥ 18 years, male and female  Histologically or cytologically confirmed hepatocellular carcinoma OR diagnosis made with characteristic enhancement in 4-Phase CT or MRI corresponding to AASLD- / EASL guidelines in cirrhotic patients.  Discussion in a routine multidisciplinary tumour board  Patients suitable for TACE  Understanding of procedure, significance and consequences of the study  signed informed consent  bilirubin has to be <2 mg/dL, AST or ALT < 6 x the upper limit of normal, international normalized ratio < 1.5 except if patients are on oral anticoagulation, haemoglobin≥ 90 g/L, platelets ≥ 50 x 109/L, and neutrophils ≥ 1.0 x 109/L |
| Exclusion Criteria |
| None of the following criteria must be present at the time of registration:  Child-Turcotte-Pugh (CTP) C liver score  Hepatic encephalopathy more than Grade 1 according to Child Pugh criteria (Appendix 3)  Active hepatitis  Gastric, duodenal or variceal bleed within 2 months of registration  Prior radiotherapy of the region to be treated  For female patients: Pregnancy, planned pregnancy |

Supp. Table 2A. QOL at baseline between the two groups

|  | SBRT  mean (std dev) | TACE  mean (std dev) | *p* |
| --- | --- | --- | --- |
| Physical functioning | 65 (± 22) | 74 (± 20) | *0.37* |
| Role functioning | 53 (± 31) | 66 (± 28) | *0.36* |
| Emotional functioning | 61 (± 27) | 63 (± 16) | *0.97* |
| Cognitive functioning | 72 (± 31) | 85 (± 15) | *0.48* |
| Social functioning | 75 (± 30) | 81 (± 24) | *0.78* |
| Global health status/QoL | 55 (±19) | 56 (± 23) | *0.88* |
| Fatigue | 51 (± 28) | 48 (± 22) | *0.88* |
| Nausea/Vomiting | 10 (± 19) | 3 (± 7) | *0.67* |
| Pain | 27 (± 33) | 25 (± 22) | *0.87* |
| Dyspnoea | 26 (± 31) | 14 (± 24) | *0.36* |
| Insomnia | 31 (± 32) | 22 (± 23) | *0.59* |
| Appetite loss | 20 (± 30) | 11 (± 16) | *0.63* |
| Constipation | 6 (± 13) | 11 (± 23) | *0.83* |
| Diarrhoea | 11 (± 20) | 11 (± 16) | *0.85* |
| Financial problems | 8 (± 19) | 22 (± 37) | *0.41* |
| Urinary frequency | 53 (± 30) | 52 (± 13) | *0.97* |
| Body image | 91 (±9) | 82 (± 25) | *0.79* |
| Blood or mucus in the stool | 98 (±4) | 95 (±22) | *0.71* |
| Stool frequency | 94 (± 10) | 83 (± 15) | *0.08* |

Std dev: standard deviation

Supp. Table 2B. Difference between QOL at baseline vs 1^st^ and 2^nd^ follow up in the SBRT group

|  | Baseline vs 1^st^ follow up  Mean (std dev) | *p* | Baseline vs 2^nd^ follow up  Mean (std dev) | *p* |
| --- | --- | --- | --- | --- |
| Physical functioning | 65 (± 22) vs 75 (± 17) | *0.59* | 65 (± 22) vs 62 (± 37) | *0.71* |
| Role functioning | 53 (± 31) vs 72 (± 31) | *0.43* | 53 (± 31) vs 59 (±42) | *0.87* |
| Emotional functioning | 61 (± 27) vs 79 (± 15) | *0.06* | 61 (± 27) vs 70 (±29) | *1.0* |
| Cognitive functioning | 72 (± 31) vs 81 (± 18) | *0.83* | 72 (± 31) vs 68 (±41) | *0.56* |
| Social functioning | 75 (± 30) vs 80 (±20) | *0.75* | 75 (± 30) vs 66 (±40) | *0.62* |
| Global health status/QoL | 55 (± 19) vs 69 (± 16) | *0.12* | 55 (± 19) vs 58 (± 27) | *0.87* |
| Fatigue | 51 (± 28) vs 33 (± 21) | *0.34* | 51 (± 28) vs 43 (± 37) | *0.71* |
| Nausea/Vomiting | 10 (± 19) vs 16 (± 30) | *0.37* | 10 (± 19) vs 12 (± 33) | *1.0* |
| Pain | 27 (± 33) vs 24 (± 31) | *0.87* | 27 (± 33) vs 14 (± 22) | *1.0* |
| Dyspnoea | 26 (± 31) vs 24 (± 30) | *0.50* | 26 (± 31) vs 33 (± 40) | *0.75* |
| Insomnia | 31 (± 32) vs 15 (± 22) | *0.62* | 31 (± 32) vs 22 (± 33) | *0.50* |
| Appetite loss | 20 (± 30) vs 15 (± 34) | *0.62* | 20 (± 30) vs 14 (± 24) | *1.0* |
| Constipation | 6 (± 13) vs 9 (± 21) | *1.0* | 6 (± 13) vs 18 (± 37) | *0.75* |
| Diarrhoea | 11 (± 20) vs 9 (± 21) | *1.0* | 11 (± 20) vs 0 (± 0) | *0.5* |
| Financial problems | 8 (± 19) vs 18 (± 27) | *1.0* | 8 (± 19) vs 0 (± 0) | *1.0* |
| Urinary frequency | 53 (± 30) vs 54 (± 26) | *0.74* | 53 (± 30) vs 70 (± 29) | *0.18* |
| Body image | 91 (±9) vs 93 (± 11) | *0.35* | 91 (±9) vs 84 (± 22) | *0.75* |
| Blood or mucus in the stool | 98 (±4) vs 96 (± 6) | *0.50* | 98 (±4) vs 85 (± 29) | *0.50* |
| Stool frequency | 94 (± 10) vs 96 (± 7) | *1.0* | 94 (± 10) vs 100 (± 0) | *1.0* |

QoL: quality of life

Supp. Table 2C. Difference between QOL before and after TACE

|  | Before vs after TACE  Mean (std dev) | *p* |
| --- | --- | --- |
| Physical functioning | 74 (± 20) vs 69 (± 22) | *0.50* |
| Role functioning | 66 (± 28) vs 61 (± 34) | *0.50* |
| Emotional functioning | 63 (± 16) vs 64 (± 19) | *0.50* |
| Cognitive functioning | 85 (± 15) vs 80 (± 27) | *0.25* |
| Social functioning | 81 (± 24) vs 76 (23) | *0.50* |
| Global health status/QoL | 56 (± 23) vs 52 (± 7) | *0.75* |
| Fatigue | 48 (± 22) vs 49 (± 20) | *0.50* |
| Nausea/Vomiting | 3 (± 7) vs 9 (± 25) | *1.0* |
| Pain | 25 (± 22) vs 40 (± 25) | *1.0* |
| Dyspnoe | 14 (± 24) vs 33 (± 19) | *1.0* |
| Insomnia | 22 (± 23) vs 23 (± 41) | *0.50* |
| Appetite loss | 11 (± 16) vs 14 (± 17) | *1.0* |
| Constipation | 11 (± 23) vs 23 (± 31) | *1.0* |
| Diarhoea | 11 (± 16) vs 5 (± 13) | *1.0* |
| Financial problems | 22 (± 37) vs 28 (± 40) | *0.5* |
| Urinary frequency | 52 (± 13) vs 61 (± 22) | *1.0* |
| Body image | 82 (± 25) vs 77 (± 34) | *0.50* |
| Blood or mucus in the stool | 95 (±22) vs 94 (± 13) | *1.0* |
| Stool frequency | 83 (± 15) vs 95 (± 12) | *0.50* |

Supp. Table 3. ALBI grade and Child-Pugh score changes during follow up

|  | SBRT | | TACE | |
| --- | --- | --- | --- | --- |
|  | ALBI grade | Child Pugh score | ALBI grade | Child Pugh score |
| Baseline, median (range)  3 months, median (range)  6 months, median (range)  9 months, median (range)  12 months, median (range) | 1 (1-2)  1 (1-3)  1 (1-3)  1 (1-3)  1 (1-2) | 6 (5-9)  5 (5-9)  5 (5-9)  5 (5-10)  5 (5-7) | 2 (1-3)  2 (1-3)  2 (2-3)  2 (2-3)  2 (1-3) | 5 (5-8)  5 (5-10)  5 (5-10)  5 (5-10)  5 (5-10) |

Supp. Table 4. Laboratory analysis in the SBRT and TACE group (baseline vs follow-up)

| SBRT | Baseline  median (range) | | Last day  median (range) | 3 months  median (range) | 6 months  median (range) | 9 months  median (range) | 12 months  median (range) |
| --- | --- | --- | --- | --- | --- | --- | --- |
| PLT [10^3^/µl]  AST[U/l]  ALT[U/l]  ALP[U/l]  bilirubin[mg/dl]  albumin[g/dl]  AFP[ng/ml] | | 169 (92-536)  66 (28-400)  48 (17-177)  192 (90-252)  0.5 (0.4-3.2)  3.8 (2.8-4.9)  317(3.8-60500) | 143 (79-249)  64 (33-364)  48 (24-87)  239 (91-478)  0.5 (0.3-3.8)  4.2 (2.6-4.7)  112(3.8-2631) | 138 (76-218)  58 (30-261)  49 (29-97)  195 (89-501)  0.5 (0.2-4.5)  4 (2.6-5.2)  15.5(3.2-85911) | 145 (70-212)  45 (29-92)  38 (17-72)  168 (91-314)  0.5 (0.3-4.2)  4.1 (2.8-4.6)  16.6 (3.9-704) | 142 (86-210)  39 (37-70)  26 (21-56)  136 (83-384)  0.5 (0.3-2.4)  4 (3.8-4.6)  47 (4.1-2908) | 137 (81-200)  45 (25-47)  30 (17-37)  140 (117-200)  0.5 (0.2-2.2)  4.1 (3.6-4.6)  22 (2.6-1755) |

| TACE | Baseline  median (range) | 3 months  median (range) | 6 months  median (range) | 9 months  median (range) | 12 months  median (range) |
| --- | --- | --- | --- | --- | --- |
| PLT [10^3^/µl]  AST[U/l]  ALT[U/l]  ALP[U/l]  bilirubin[mg/dl]  albumin[g/dl]  AFP[ng/ml] | 121 (64-355)  63 (26-2404)  31 (20-1050)  111 (67-188)  1.2 (0.5-2.6)  3.8 (2.7-4.8)  5.1 (2.7-11.4) | 156 (63-273)  52 (35-1099)  31 (20-734)  125 (80-336)  1 (0.5-16.9)  3.8 (3.2-4.3)  4.3 (1.9-15.2) | 145 (62-222)  60 (24-234)  31 (13-360)  220 (78-294)  1.2 (0.6-9.8)  3.5 (2.6-3.6)  6.3 (2.3-15.2) | 103 (77-229)  66 (30-195)  51 (24-98)  256 (256-256)  1.3 (0.5-12.3)  3.8 (2.8-4.5)  4 (1.1-24046) | 95 (74-272)  77 (32-1019)  48 (35-469)  164 (83-245)  1.4 (0.5-7)  3.5 (2.8-41)  33 (5.2-159) |

PLT, platelets; AST, aspartat aminotransferase; ALT, alanine aminotransferase; ALP, Alkaline phosphatase ; AFP, alpha phetoprotein
