# Supplementary figures and images for: Efficacy of Stereotactic Body Radiotherapy in Patients With Hepatocellular Carcinoma Not Suitable for Transarterial Chemoembolization (HERACLES: HEpatocellular Carcinoma Stereotactic RAdiotherapy CLinical Efficacy Study)
Source: Front Oncol. 2021 Mar 19;11:653141. doi: 10.3389/fonc.2021.653141 (PMC8017336; doi:10.3389/fonc.2021.653141)

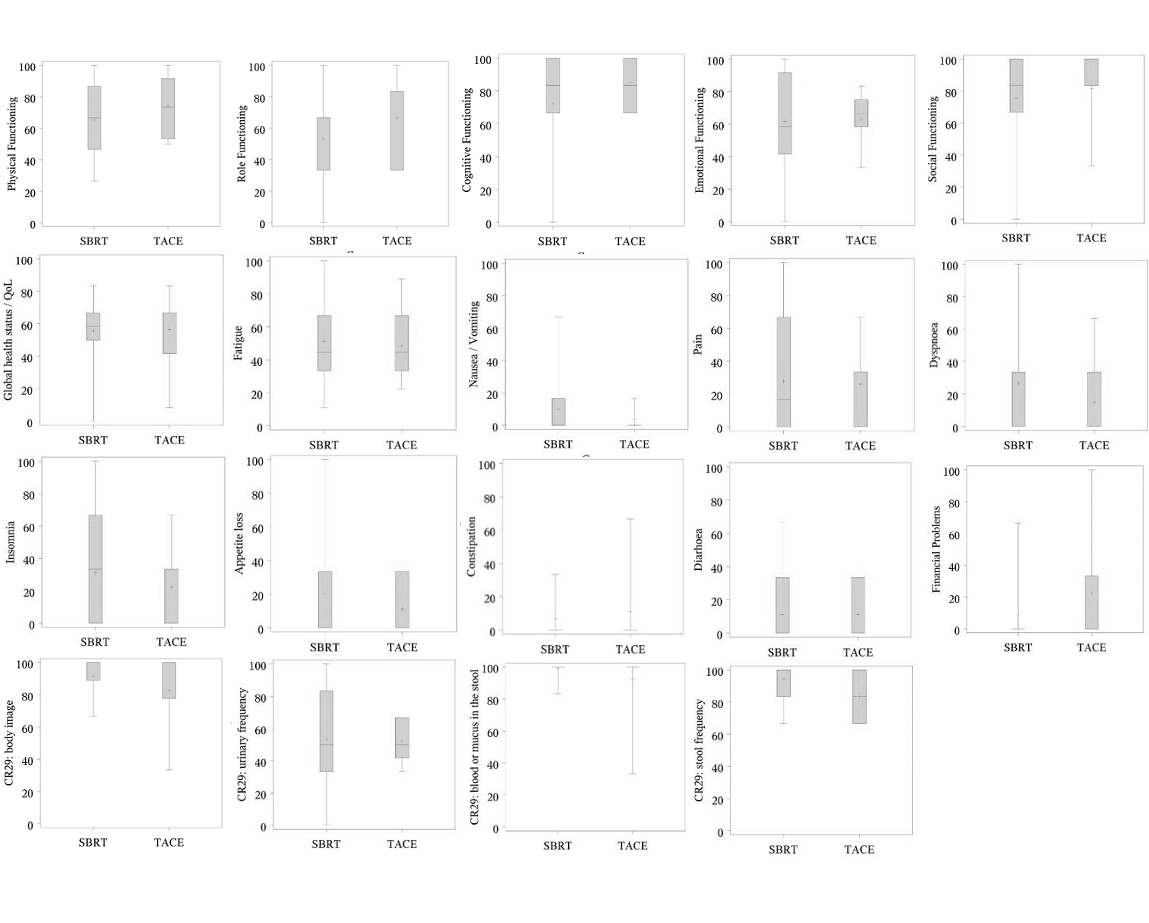

Supplement: Supplementary file 2 [file Image_1.TIF]
